# Modeling Light Adaptation in Circadian Clock: Prediction of the Response That Stabilizes Entrainment

**DOI:** 10.1371/journal.pone.0020880

**Published:** 2011-06-16

**Authors:** Kunichika Tsumoto, Gen Kurosawa, Tetsuya Yoshinaga, Kazuyuki Aihara

**Affiliations:** 1 Aihara Complexity Modelling Project, ERATO, Japan Science and Technology Agency, Tokyo, Japan; 2 Institute of Health Biosciences, University of Tokushima, Tokushima, Japan; 3 Institute of Industrial Science, University of Tokyo, Tokyo, Japan; Vanderbilt University, United States of America

## Abstract

Periods of biological clocks are close to but often different from the rotation period of the earth. Thus, the clocks of organisms must be adjusted to synchronize with day-night cycles. The primary signal that adjusts the clocks is light. In *Neurospora*, light transiently up-regulates the expression of specific clock genes. This molecular response to light is called light adaptation. Does light adaptation occur in other organisms? Using published experimental data, we first estimated the time course of the up-regulation rate of gene expression by light. Intriguingly, the estimated up-regulation rate was transient during light period in mice as well as *Neurospora*. Next, we constructed a computational model to consider how light adaptation had an effect on the entrainment of circadian oscillation to 24-h light-dark cycles. We found that cellular oscillations are more likely to be destabilized without light adaption especially when light intensity is very high. From the present results, we predict that the instability of circadian oscillations under 24-h light-dark cycles can be experimentally observed if light adaptation is altered. We conclude that the functional consequence of light adaptation is to increase the adjustability to 24-h light-dark cycles and then adapt to fluctuating environments in nature.

## Introduction

Organisms have evolved to have internal biological clocks in a response to the environmental cycles of days and nights. In most eukaryotes, transcriptional and translational feedback regulations have been suggested to underlie circadian oscillations in the abundance of mRNAs and proteins [1]. The period of biological clocks under constant conditions is not exactly 24 h. Therefore, these clocks adapted to synchronize with 24 h environmental cycle. Experimental works [2]–[4] have reported that there were some cases of dysfunction of synchronization such as so-called relative coordination. Relative coordination is a phenomenon such that circadian rhythms are not entrained by external cycles and oscillations that co-exist with internal and external periods can appear, and that is thought to be related with sleep disorder in human [1], [5], [6].

Experimental advances have revealed much information underlying adaptation mechanisms in a response to the environmental cycles as well as mechanisms for the generation of oscillation (1). Light up-regulates the expression of white collar-1 (*wc-*1) and white collar-2 (*wc-*2) genes in *Neurospora* that causes frequency (*frq*) gene expression to activate [7]–[13]. Indeed, the amount of *frq* gene transcript reaches about 10 times that under constant darkness (DD) conditions within 30 min [10]. Similarly, light up-regulates the expression of *period*1 (*per*1) and *period*2 (*per*2) genes in mammals [14]–[18]. Switching from low-activation state to high-activation state of gene expression correlated with a transition from dark to light may underlie the entrainment in *Neurospora* and mammals.

One approach toward understanding the entrainment of circadian rhythms is mathematical modeling [19]–[30]. Modeling predicts autonomous oscillations can be entrained by light-dark (LD) cycles in which negative feedback regulation on gene expression and induction of gene by light are assumed [28].

However, above-mentioned induction of genes by light does not continue for many hours under constant light [8]–[15]. This molecular response to light is called light adaptation [1], [12], [13]. Instead, the transcript of light-induced genes such as *frq*, *wc*-1, and *vivid* (*vvd*) genes in *Neurospora* starts to decrease and it reaches the same as that under DD conditions within one hour even in the continued presence of light [8]–[13]. Similarly, the induction by light of the *per*1 gene in mammals seems to be transient. Exposure to light for 30 min can sharply increase the abundance of *per*1 mRNA [14], [15]. Subsequently, the increase in *per*1 mRNA ceases and a decrease in *per*1 mRNA is observed within one hour after exposure to light has terminated. Under DD conditions, after *per*1 mRNA has accumulated, the PER1 protein is known to peak with a lag of four hours [18], [31]. Therefore, the decrease in *per*1 mRNA within one hour after exposure to light has terminated is probably not due to negative feedback on its own transcription by the PER1 protein. Although there is no direct evidence that the light induction of the *per*1 gene in mammals is transient under constant light, some studies have shown that activation of MAPK signaling by light, which is a key intracellular pathway that links light to the gene expression, is transient during constant light [32]–[35]. Thus induction of *per* gene by light via MAPK signaling might be also transient. To our knowledge, function of light adaptation in synchronization with environmental cycles of days and nights remains unclear while several studies have focused on molecular mechanisms underlying adaptation [1], [13], [36]–[39].

In this article, we first estimated molecular response to light using a mathematical model and experimental data, and found that estimated up-regulation of genes by light is transient during light period in mouse as well as *Neurospora*. Then, we constructed a mathematical model to examine the function of transient response to light in synchronization with LD cycles. We found that circadian oscillations are more likely to be entrained by 24-h LD cycles when up-regulation of gene by light is transiently repressed. The results of the present study indicate the possibility that the functional consequence of the transient repression of gene expression by adaptation to light is to enhance the entrainability of circadian rhythms under LD cycles that have various light intensity in nature.

## Results

### A mathematical model of circadian clock incorporating light adaptation

**Figure 1:**
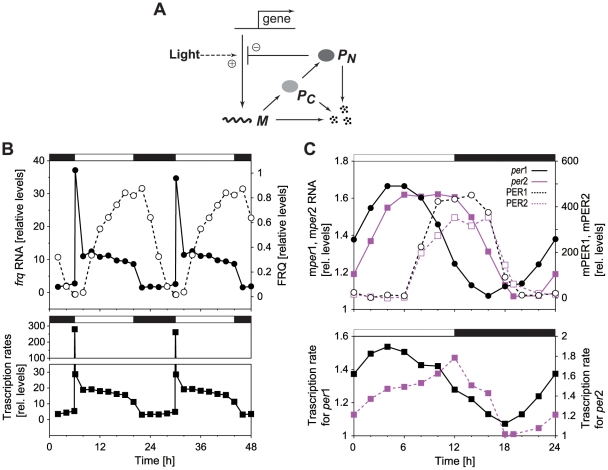
Estimated light response using experimental data and model. A schematic diagram of the minimal clock model involving a single auto-regulated gene in which light up-regulates the transcription (A), estimated temporal change of transcription rate of a clock gene under light-dark (LD) cycles from experimental data of *Neurospora* (B) and mouse (C). Relative abundances of *frq* RNA (closed symbols in upper panel of (B)) and FRQ protein (open symbols), and m*Per* RNAs (closed symbols in upper panel of (C)) and PER proteins (open symbols) were redrawn from the published experimental data [10], [18]. Using the experimental data and equations (1–3), the temporal variations of the transcription rate of *frq* mRNA in *Neurospora* and those of m*Per*1 and m*Per*2 mRNAs in mouse were estimated (lower panel of (B) and (C)). The LD cycles are represented by the white and black bars, respectively.

To study what effect light adaptation has on circadian oscillations, we consider a mathematical model for a circadian clock [22] as simple as possible based on a limit cycle oscillator (Figure 1A) and a case in which the expression of a clock gene is up-regulated by light. We assume that the dynamics of the cellular circadian oscillation is governed by
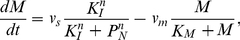
(1)

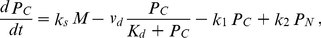
(2)

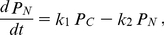
(3)where *M*, *P_C_*, and *P_N_* correspond to the concentration of mRNAs, protein in cytosol and that in the nucleus. The mRNAs are synthesized according to a Hill equation with the amount of the protein, *P_N_*, in the nucleus. The 2nd term of Eqs. 1 and 2 indicates enzymatic degradation of mRNAs (*M*) and proteins (*P_C_*). Negative feedback regulation of gene expression by nuclear inhibitor (*P_N_*) is required for the generation of circadian rhythms for many species such as *Drosophila*, mammals and *Neurospora*. The period of autonomous oscillation observed in Eqs. 1–3 varies from 20 h to 27 h as the transcription rate, *v_s_*, increases from 1.3 nM/h to 3.2 nM/h. For the choice of parameter value, *v_s_* = 1.6 nM/h, limit cycle oscillations with a period of 21.5 h corresponding to the endogenous period of wild-type *Neurospora* under DD conditions can be obtained by Eqs. 1–3 [22], [28]. The model can also generate autonomous oscillation with a period of 25 h, which corresponds to the endogenous period of mammals under DD condition, by changing the parameter of transcription rate *v_s_* in Eq. 1 to 2.5 nM/h. Molecular systems for circadian rhythms are composed of complex networks with transcriptional/translational feedback regulations [1], [37], [40]. Therefore, many other genes may underlie the generation of circadian oscillations though we disregard these for the sake of simplicity.

Meanwhile, light up-regulates the transcription of clock gene in *Neurospora* and mammals [7], [14], [41] while light down-regulates protein stability in *Drosophila* [42], [43]. In this study, we incorporated the effect of LD cycles as temporal changes in the transcription rate, *v_s_*, into the autonomous circadian oscillator model. We herein introduce a variable *X* as transcriptional response to light that is defined as an increased ratio of transcription rate under the light phase to that under the dark phase. The transcription rate *v_s_* in Eq. 1 is replaced with *v_s_*(1+*X*) for the calculation of circadian oscillations under LD cycles.

### Estimation of light response using experimental data and model

In *Neurospora*, light induces *frq* mRNA via acute increase in the White Collar Complex (WCC), which is a heterodimer of WC-1 and WC-2, followed by an immediate reduction in *frq* mRNA, which may be interpreted as an increase and subsequent decrease in transcription rate *v_s_* in Eq. 1 [10]. Indeed, not only up-regulation by light but also negative feedback regulation and the degradation simultaneously affect the abundance of mRNAs. Thus, we need to separate these effects in order to estimate up-regulation by light. In order to know whether temporal variation of the transcription rate *v_s_* changes under an LD cycle, we first estimated parameters except for *v_s_* using experimental data under DD condition [44] and Eqs. 1–3. Then, temporal variation of *v_s_* was estimated using the time series data of *frq* mRNA and FRQ by Tan et al. [10] and Eqs. 1–3 (Figure 1B). The estimated transcription rate for *frq* acutely increases, reaches a maximum within 1 h after the lights on, and immediately decreases, approaching a plateau that is low but significantly higher than the rate in the dark. Subsequently, the transcription rate gradually decreases by the level of that in the dark (lower panel in Figure 1B). Thus, up-regulation of *frq* gene by light was estimated to be light adaptation. In addition, the transcription rates for m*per*1 and m*per*2 genes in mouse under an LD cycle were estimated by using experimental data of m*Per*1 mRNA, m*Per*2 mRNA, mPER1, and mPER2 in mouse SCN by Field et al. [18] and Eqs. 1–3. Notably, using the parameters except for *v_s_*, which are estimated from DD data in SCN [45], *v_s_* cannot be estimated for LD data if we assume *v_s_*, is constant during light period. The estimated transcription rate for m*Per*1 mRNA increases promptly after the light on, and peaks at 4 h after exposure to light (Figure 1C). Subsequently, light induction of m*Per*1 mRNA is followed by the suppression of the induction even in the presence of light. Thus, up-regulation of m*per*1 gene was estimated to be also light adaptation. In contrast, the transcription rate for m*Per*2 mRNA increases more slowly from the onset of the light phase. After reaching the maximum value at the offset of the light phase, the transcription rate rapidly decreases. Interestingly, the estimated transcription rate for m*Per*1 and m*Per*2 mRNAs at dark period is not constant whereas that for *frq* mRNA in *Neurospora* is almost constant at dark period. It is probably because detailed posttranslational regulation of mPER1 and mPER2 including complex formation with CRYs is neglected in this estimation for the sake of simplicity.

**Figure 2:**
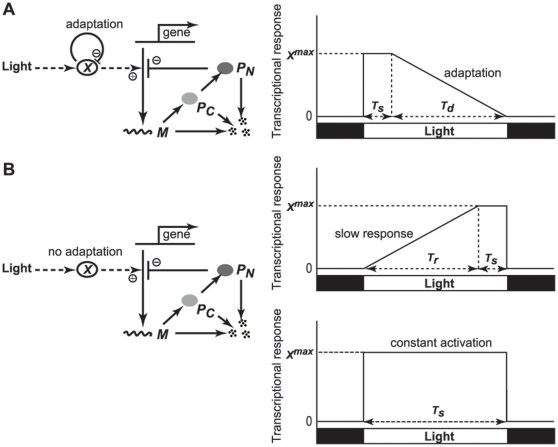
Schematic diagrams for circadian oscillator with light adaptation. Three kinds of light induction were theoretically considered that are up-regulation of transcription by light in the form of light adaptation (A), slow response, and no adaptation (B). In the case of light adaptation, the up-regulated transcription rate remains at a maximal value, *X^max^* for a certain period with duration *T_s_*. Subsequently, the transcription rate decreases over a decay time, *T_d_*, and reaches to the basal transcription rate under dark (A). In the case of slow response, the up-regulated transcription rate increases over a period *T_r_* and reaches to the maximum (B).

In this study, we theoretically consider three kinds of light induction as depicted in Figure 2: (I) transcription rate is up-regulated by light and is subsequently reduced (light adaptation); (II) the rate is elevated, and this up-regulation continues to be constant during light period (no adaptation); (III) the rate gradually increases during light period (slow response). Results of the estimated transcription rates indicate that light induction of *frq* gene in *Neurospora* and m*per*1 gene in mouse is the type (I) and that of m*per*2 gene is the type (III). Figure 2A illustrates the case for the abrupt increase in transcriptional response at the dark-light transition followed by a gradual decrease. After the dark-light transition, the transcriptional response, *X*(*t*), remains at the highest constant value of *X^max^* for a certain period with duration *T_s_*. Subsequently the transcriptional response *X*(*t*) linearly decreases over a decay time, *T_d_*, and reaches zero (*X(t)* = 0), corresponding to the basal transcription rate, *v_s_*, under dark phase. Temporal change of transcriptional response *X(t)* to light is defined explicitly as
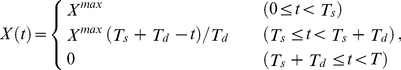
(4)where *T* represents the period of the LD cycle, *T_s_* is the duration, and *T_d_* is the decay time. Duration *T_s_*+*T_d_* is set to be smaller than or equal to that of light period *T*/2. In the present study, varying *T_s_* or *T_d_*, or both, we construct variations in transcriptional response to light and determine the types of response, *X(t)*, that stabilize or destabilize entrainment under LD cycles.

### Light induction followed by reduction of mRNA increases entrainability

**Figure 3:**
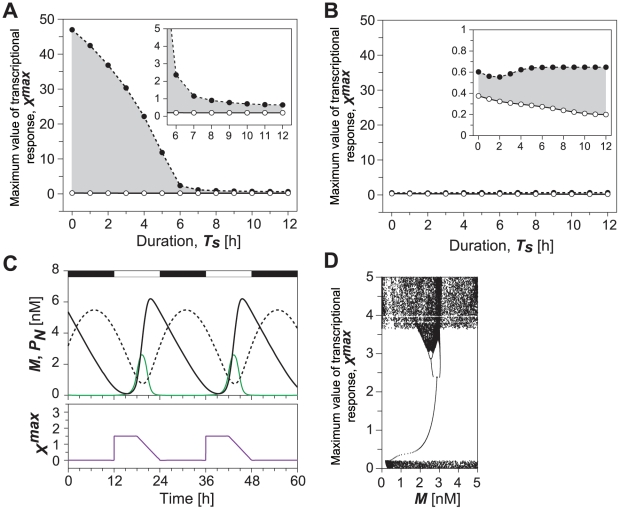
Effects of light adaptation on entrainment of circadian oscillations. We observed dynamic behavior as a function of the maximum transcriptional response (*X^max^*) and duration of maximum response (*T_s_*) when up-regulation of transcription by light is in the form of light adaptation (A) and slow response (B). The lower (open circles) and upper (closed circles) limits correspond to the saddle-node and the period-doubling bifurcation points. Gray shading in A and B indicates condition for the circadian oscillations entrained by 12 h∶12 h LD cycles. In the calculation, *T_s_*+*T_d_* and *T_s_*+*T_r_* were fixed as 12 h. The insets in A and B are enlarged diagrams in a certain range of duration *T_s_*. (C) Entrained oscillation of mRNA (black solid) and nuclear protein (dashed) when up-regulation of transcription by light is in the form of light adaptation (lower panel). The time course for mRNA production is also depicted (Green). (D) A one-parameter bifurcation diagram of attractors as a function of the maximum response, *X^max^*. Parameter values in Eqs. 1–3 are: *n* = 4, *K_I_* = 1 nM, *v_s_* = 1.6 nM/h, *v_m_* = 0.505 nM/h, *K_M_* = 0.5 nM, *k_s_* = 0.5 h^−1^, *v_d_* = 1.4 nM/h, *K_d_* = 0.13 nM, *k*_1_ = 0.5 h^−1^, and *k*_2_ = 0.6 h^−1^. *T_s_* = *T_d_* = 6 h (C, D) and *X^max^* = 1.5 (C).

Figure 3A shows the entrainment range for circadian oscillation under 12 h∶12 h LD cycles when the maximum value of transcriptional response, *X^max^*, and duration *T_s_* of the maximum transcriptional response during the light phase are varied. Here, decay-time *T_d_* is also changed implicitly, because we assume that the time that the transcriptional response takes to return from the maximum to zero agrees with the offset of the light phase in the LD cycle (i.e. *T_s_*+*T_d_* = 12 h). Thus, the case where the duration of maximum transcriptional response, *T_s_* is 12 h corresponds to a square-wave variation in transcriptional response to LD cycles. In Figure 3A, there exist upper and lower limits of entrainment region for the value of *X^max^*. The upper limit for entrainment drastically increases as duration *T_s_* of maximum transcriptional response decreases while the lower limit hardly changes due to variations in duration *T_s_*. When duration *T_s_* is less than about 6 h, the upper limits of the entrainment range expands by about fifty times more than that of the square-wave. Hence, this suggests that if it causes an abrupt decrease in the transcriptional response, the transcriptional response at the offset of the light phase (light-dark transition) decreases the entrainability of circadian oscillations under LD cycles.

We can observe various complex oscillations beyond the lower and the upper limits of the entrainment range. The lower limit corresponds to a saddle-node bifurcation and the upper to a period-doubling one. For example, when the value of *X^max^* is very small, the autonomous circadian oscillation with the period of 21.5 h under DD cannot be entrained to 24-h LD cycles. Then, the response of the autonomous oscillation to the LD cycles is quasi-periodic (Figure 3D and S1A), which is a type of oscillation executed by a dynamical system containing a finite number (two or more) of incommensurable frequencies. In Figure 3A, a saddle-node bifurcation is caused by passing through the lower limits with an increase of the value of *X^max^*. After the occurrence of the saddle-node bifurcation, an oscillation entrained by LD cycles appears (Figure 3C). However, we can see that the stable entrained oscillation becomes unstable when the value of *X^max^* increases across the upper limit in Figure 3A. Then, the stable oscillation changes to a period-2 oscillation due to the occurrence of the period-doubling bifurcation of the entrained oscillation. Moreover, a cascade of period-doubling bifurcations can be observed as the value of *X^max^* further increases (Figure 3D). Consequently, a chaotic oscillation occurs (e.g., see Figure S1B).

It still remains possible that an abrupt increase in the transcriptional response at the onset of the light phase, i.e., dark-light transition, also decreases entrainability. To test this possibility, we consider a case where there is an abrupt decrease in the transcriptional response at the offset of the light phase preceded by a gradual increase in the transcriptional response. Figure 2B depicts such a transcriptional response, *X*(*t*), in which *X*(*t*) linearly increases over the rise time, *T_r_*, and reaches the maximum value, *X^max^*, earlier than the offset of the light phase. Subsequently, the transcriptional response *X*(*t*) is sustained at a highest constant value, *X^max^*, for duration *T_s_* and it decreases abruptly at the offset of the light phase. As shown in Figure 3B, the variations in duration *T_s_* have no apparent effect on critical value for saddle-node and period-doubling bifurcation. This result indicates that the dark-light transition at the onset of the light phase does not affect the entrainability of circadian oscillations under LD cycles. For comparison, we investigated the effect of both light adaptation and slow response on the entrainability of circadian oscillation with a period of 25 h. As shown in Figure S2A and S2B, we found that the tendency was essentially the same as Figure 3A and 3B.

Previously, Gonze and Goldbeter have demonstrated that if the temporal changes in the transcription rate were altered from a square to a sinusoidal waveform, circadian oscillations were more likely to be entrained by 12∶12 LD cycles [28]. These results indicated the possibility that the abrupt change in the transcription rate under LD cycles decreased entrainability. However, it still remained unclear whether the abrupt increases of the transcriptional response at the dark-light transition or the abrupt decreases of that at the light-dark transition decreases entrainability. Together with the result shown in Figure 3A, we can conclude that the abrupt decrease in the transcriptional response at the offset of the light phase decreases the entrainability of circadian oscillations under LD cycles while its abrupt increase at the onset of the light phase hardly affects it. In other words, these results (Figure 3A and 3B) suggest that the entrainability under LD cycles increases if up-regulation of gene by light is transiently repressed during light period as observed in *frq* gene of *Neurospora* and m*per*1 gene of mouse (Figure 1B, 1C, and 2A). In contrast, slow transcriptional response to light, which is observed in m*per*2 gene in mouse (Figure 1C and 2B), has little effect on the entrainability of circadian oscillations under LD cycles.

### Slow repression after light induction increases entrainability

Now, two possibilities may arise underlying the increase in entrainability when light induction is followed by reduction of mRNA. The shorter duration of the maximum transcriptional response increases entrainability or the slow repression of the induction following the maximum transcriptional response increases the entrainability of circadian oscillations under LD cycles.

**Figure 4:**
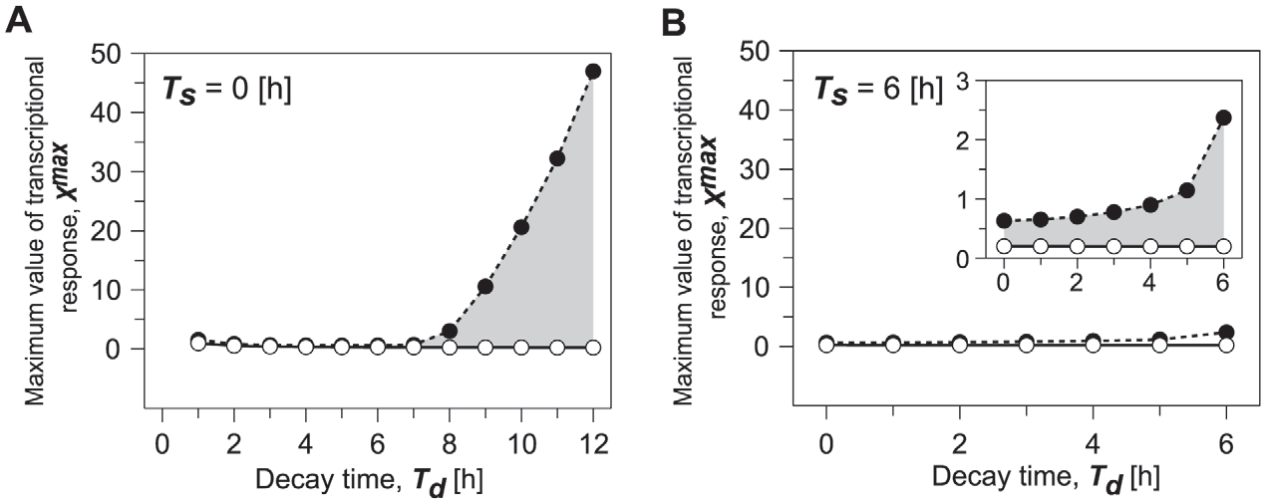
Effects of decay time during light adaptation on entrainment of circadian oscillations. While duration of maximum transcriptional response to light, *T_s_* was fixed as 0 h (A) and 6 h (B), we observed dynamic behavior as a function of the maximum response (*X^max^*) and the duration of decay time (*T_d_*). The inset in B is enlarged diagrams in a certain range of decay-time *T_d_*. Gray shading in A and B indicates condition for the circadian oscillations entrained by 24-h light-dark cycles. The open and closed circles correspond to saddle-node and period-doubling bifurcation points. Parameters except for *T_s_* and *T_d_* are the same as in Figure 3.

To verify these possibilities, we investigated what effect variations in decay-time *T_d_* of transcriptional response would have on the entrainment range when duration *T_s_* of maximum transcriptional response was fixed. In Figure 4A, duration *T_s_* is set to zero, implying that the transcriptional response is instantaneously increased up to *X^max^* and decay-time *T_d_* is varied, implying that it decreases linearly for *T_d_* hours and reaches zero. Thus, the temporal variations in the transcriptional response with *T_d_* = 12 h in Figure 4A are the same as those with *T_s_* = 0 h in Figure 3A. The upper and lower limits correspond to period-doubling and saddle-node bifurcations. The basin surrounded by these limits yields a parameter set, giving rise to circadian oscillations entrained by LD cycles. When duration *T_s_* of the maximum transcriptional response is equal to zero and decay-time *T_d_* is less than 7 h, the region of entrainment remains unchanged. However, if decay-time *T_d_* is longer than 7 h, the region of entrainment enlarges as decay-time *T_d_* increases (Figure 4A). Even though duration *T_s_* was varied, we always observed that the increase in decay time tended to enlarge the entrainment region (Figure 4B). Thus, the slow suppression of the induction following maximum transcriptional response increases the entrainability.

**Figure 5:**
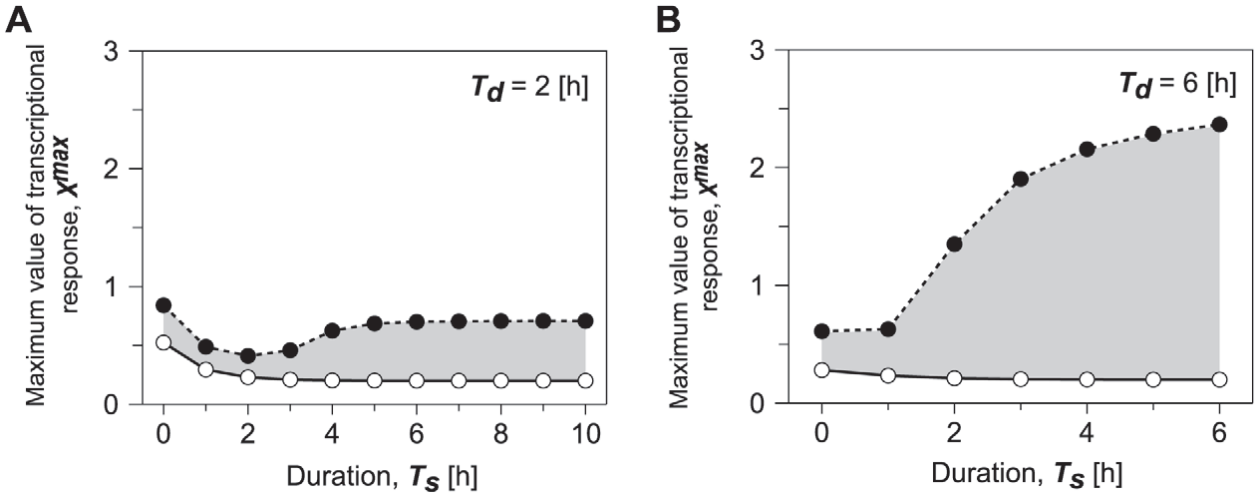
Effects of maximum transcriptional response during light adaptation on entrainment of circadian oscillations. While duration of decay time during light adaptation, *T_d_* was fixed as 2 h (A) and 6 h (B), we observed dynamic behavior as a function of the maximum response (*X^max^*) and the duration of maximum transcriptional response (*T_s_*). Gray shading in A and B indicates condition for the circadian oscillations entrained by 24-h LD cycles. The open and closed circles correspond to saddle-node and period-doubling bifurcation points. Parameters except for *T_s_* and *T_d_* are the same as in Figure 3.

We next fixed decay-time *T_d_* and varied duration *T_s_* (Figure 5). When decay-time is fixed e.g., 2 h or less, which corresponds to the fast repression in transcriptional response, the change in duration *T_s_* does not greatly alter entrainability (Figure 5A). Surprisingly, the region of entrainment enlarges but does not greatly change as duration *T_s_* increases, when decay-time *T_d_* was fixed at 6 h or more, which is the slow suppression in transcriptional response (Figure 5B). These results provide the reason why entrainability of circadian oscillations under LD cycles increases when light adaptation is incorporated (Figure 3A). Thus, not the decrease in *T_s_* but the increase in decay-time *T_d_*, i.e., the slow suppression in transcriptional response increases the entrainability. In order to test the generality of this conclusion, we numerically analyzed the model for other parameter sets, and we confirmed that this conclusion always holds for all the parameter sets we studied.

### Phase response curves

We next sought to obtain the conditions for the occurrence of oscillations entrained to environmental cycles by analyzing the magnitude of phase shifts induced by a stimulus that depended on the timing, the strength, and the duration of the stimulus, that is phase response curve (PRC) analysis. The PRC is defined as
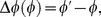
(5)where 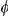 denotes the phase when a single light pulse is applied to the circadian oscillator, and 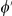 represents the phase after phase shift induced by the single light pulse. From the relationship between phase 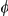 and phase-shift 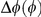, we can derive a phase transition curve (PTC), i.e., the curve when plotted as new phase 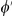 versus old phase 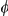 [1], [46]–[48]

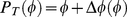
(6)When the light pulses are applied every *T* time to the circadian oscillator with the free-running period, 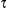, the following one-dimensional mapping can be defined as [48], [49]

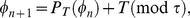
(7)where 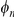 denotes the phase right before the time of the *n*th light pulses and 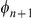 represents the new phase just before the next pulses. If there is an entrained oscillation, then Eq. 7 must satisfy 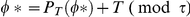 for some value of 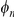. Now, let us assume that the light pulse is applied to the circadian oscillator at phase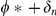, where 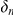 is a phase lag. If the oscillation is stable, the phase starting from phase 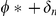 always converges to phase 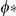 with time. According to the theory of a nonlinear dynamical system [50], the stability of the solution in Eq. 7 can be evaluated from the following linearized difference equation:
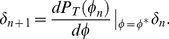
(8)If Eq. 8 satisfies condition 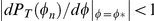, then solution 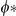 in Eq. 7, i.e., oscillation, is stable [51]. Thus, taking Eq. 6 into consideration, the stable condition for oscillation can be obtained as
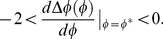
(9)


Now, let us assume that for the parameter set in Eqs. 1–3, the circadian system has a self-sustained oscillation with a period (i.e., free-running period 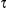) of 21.5 h in DD. If the circadian oscillation with the period of 21.5 h entrains to the external cycles with a 24-h period, the minimum phase shift must be equal to or less than −2.5 h. Therefore, the necessary condition for entrainment is that the phase shift in Eq. 5 satisfies
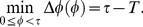
(10)From Eqs. 9 and 10, we can derive the critical conditions at which the oscillation is able to entrain to the periodic pulse train using the slope of PRC at the phase yielding 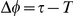 shift after the pulse. The critical conditions are:
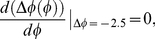
(11)

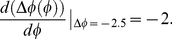
(12)Note that Eqs. 11 and 12 correspond to the necessary and sufficient conditions for saddle-node and period-doubling bifurcations.

**Figure 6:**
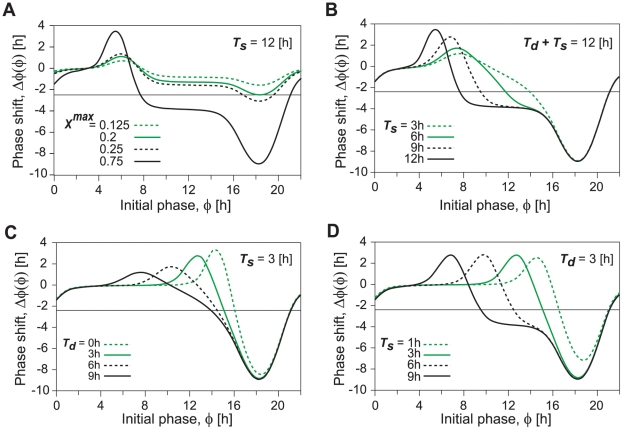
Phase responses to a single light pulse in a circadian oscillator with light adaptation. Phase responses are depicted as a function of the initial phase, the maximum transcriptional response, *X^max^* to light (A), and the duration of maximum transcriptional response during light adaptation, *T_s_* (B) when *T_s_*+*T_d_* was fixed as 12 h. The initial phase of 0 h is defined as the time yielding the minimum value of *frq* mRNA oscillations. Solid line parallel to the horizontal axis corresponds to the necessary condition for the entrainment by 24-h LD cycles (i.e. −2.5 h (free-running period – 24 h)). (C) Phase response as a function of initial phase and the duration of decay time, *T_d_* during light adaptation when duration of maximum response (*T_s_*) was fixed. (D) Phase response as a function initial phase and duration of maximum response, *T_s_* when the duration of decay time (*T_d_*) was fixed. Parameters are the same as in Figure 3. *X^max^* = 0.75 (B–D).

Figure 6A plots PRCs when light pulses having a rectangular form are applied for 12 h with intensities of *X^max^*, corresponding to the maximum value in transcriptional response, to the autonomous circadian oscillator with various initial phases. When the highest value of *X^max^* is equal to 0.125, corresponding to a weak light pulse, the oscillation with 21.5 h periods cannot be entrained to LD cycles with the period of 24 h because phase shift is always larger than −2.5 h. By increasing light intensity, the minimum value of phase shift decreases and contacts the horizontal line that represents the phase shift of −2.5 h at the light intensity i.e., *X^max^* = 0.2. Then, the entrained condition in Eq. 11 is satisfied because the slope of PRC at the contact points is equal to zero. The value of *X^max^* agrees with the maximum value in the transcriptional response when saddle-node bifurcation occurs. When the value of *X^max^* is larger than 0.2, circadian oscillations can be entrained to the LD cycles because the minimum phase shift is less than −2.5 h. Furthermore, increasing the value of *X^max^*, the slope of PRC at 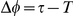 becomes steeper and can be less than −2, which corresponds to the critical point for period-doubling bifurcation given by Eq. 12. Actually, the slope of PRC at 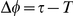 is −2.023 at *X^max^* = 0.75 and period-4 oscillation was observed under LD cycles (Figure S3).

Next, let us consider what effect the shape of light pulses has on the slope of PRCs at 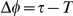. Figure 6B plots the dependence of the slope of the PRC at 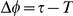 on the period for maximized response *T_s_*, when the length of the light period is fixed (i.e., *T_s_*+*T_d_* = 12 h). As the period for maximized response *T_s_* decreases from *T_s_* = 12 h, minimum phase shift remains unchanged but maximum phase shift decreases (Figure 6B). As a result, with a decrease in duration *T_s_*, the slope of PRC at 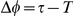 can be more than −2. In fact, the slope of the PRC at 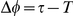 is −1.602 for *T_s_* = 9 h at *X^max^* = 0.75 and Eq. 9 is satisfied. For 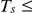
9 h at *X^max^* = 0.75, the parameter sets are within the entrained region under LD cycles (Figure 3A). In other words, when the period for maximized transcriptional response *T_s_* shortens, the occurrence of period-doubling bifurcation is suppressed for which entrained oscillation remains stable against the increase in the maximum value of *X^max^* in the transcriptional response. In Figure 6C, if duration *T_s_* is fixed at a certain period, e.g., 3 h, and decay-time *T_d_* is increased, i.e., the transcriptional response is repressed more slowly, the maximum amplitude of phase shift decreases while its minimum remains unchanged. As a result, the slope of PRC at 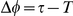 becomes smaller with the increase in *T_d_*. In contrast, Figure 6D shows that if decay-time *T_d_* is fixed, e.g., 3 h, and duration *T_s_* is increased, the slope of PRC at 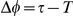 hardly changes. Therefore, the period for reduction after induction of transcription has a larger effect than the period for maximized transcriptional response on the slope of PRC at 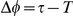.

The reason for the increase in the entrainability by slow repression after light induction of gene (i.e. the reduction of the slope of PRC at 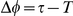 in Figure 6C) can be intuitively understood as follows. Acute reduction of transcription at the light offset can advance the phase of oscillation due to decreasing mRNA as long as mRNA decreases at the timing of light offsets. If repression after light induction occurs more slowly, effect of phase advance due to light offset becomes smaller. Furthermore, the slower suppression of the up-regulation of transcription can increase the synthesis of mRNAs and as a result it increases the abundance of nuclear proteins, functioning as inhibitor for the transcription. Increase of inhibitor proteins causes phase delay by retarding the release from negative regulation of the transcription. Thus, slower suppression following light induction tends to result in the reduction in the magnitude of phase advance in PRCs (Figure 6C). By reduction in magnitude of phase advance, the slope of PRC at 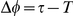 becomes more gradual that corresponds to the reduction in the occurrence of period-doubling bifurcation.

## Discussion

Phasic responses to the onset (dark-light transition), and/or the end of pulses (light-dark transition), which reset instantaneously only phase without affecting a parameter of the system, and/or tonic responses to the light in between, which modulate angular velocity by changing a parameter in the oscillator, have been thought to be processes underlying the entrainment of circadian rhythms [1], [52]–[54]. However, we still do not know which are the key elements in entrainment. By incorporating three elements into the mathematical model showing limit cycle oscillation under constant condition, we considered what effects these would have on entrainment under LD cycles. We found that light-dark transition with large forcing strength destabilizes entrainment under LD cycles while dark-light transition has almost no effect on entrainment (Figure 3A and 3B). These results clearly indicate that phasic response to the end (light-dark transition) other than the onset (dark-light transition) becomes one of the key elements in the stability of entrainment for species such as mammals and fungi in which light up-regulates the expression of clock genes. For the tonic responses to light in between, we considered the functional consequence of the transient up-regulation of specific genes by light, which was estimated using our model and published experimental data for *Neurospora* and mouse. The results in Figure 3A indicate that circadian oscillations are more likely to be entrained under LD cycles with fluctuating light intensity if the expression of the clock gene up-regulated by light is not continuous but transient. We thus concluded that tonic responses can also be one of the key elements in entrainment.

Previous theoretical studies of circadian rhythms showed that forcing strength, frequency mismatch, limit cycle amplitude, and memory of perturbations play important roles in the entrainment of circadian oscillations to the environmental cycles [28], [55], [56]. If only these factors affect the entrainment range, we expect that the parameter space for the entrainment should be the same regardless of the shape of up-regulation by light as long as *T*_s_+*T*_d_ = *T*_r_+*T*_s_ for example. However, the results indicated in Figure 3A and 3B showed that duration of activation of light induction (*T*_r_) and its suppression (*T*_d_) affects the entrainment differently.

There have been reports of so-called relative coordination in experiments in which circadian oscillations are not entrained by 24-h LD cycles. A phenomenon called relative coordination in these reports can be regarded as quasi-periodic oscillations in nonlinear dynamical systems [50]. Quasi-periodic oscillations generally have multiple frequency components in which the ratio between these frequencies is an irrational number [5]. The occurrence of quasi-periodic oscillations can be clearly understood by analyzing PRCs. The absolute value of maximum or minimum phase shifts in circadian oscillations tends to increase as the intensity of up-regulation by light increases (Figure 6A). When the intensity of up-regulation is very small, the absolute value of maximum (minimum) phase shift is smaller than 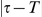 (See Eq. 10) and then, relative coordination can be observed (Figure S1A). When the intensity of up-regulation is sufficiently large, since the absolute value of maximum (minimum) phase shift is larger than 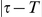, circadian oscillations can be entrained by external cycles. However, if the high intensity of up-regulation by light results in a steep slope for PRCs at 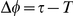 (i.e., smaller than −2) (Figure 6A), entrained oscillations are destabilized due to the occurrence of period-doubling bifurcation. Then, oscillations with higher-order periods, e.g., period-2 and period-4, can be observed (Figure 3D and S3). As well as the high intensity of up-regulation, we found that an abrupt termination of up-regulation at light offset can also destabilize entrained oscillations. In fact, if up-regulation persists during the light phase, the slope of PRCs at 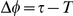 is steeper than that for transient up-regulation (Figure 6B).

As light intensity increases, phase shift in behavioral rhythm has been reported to become larger (1). We predict that, from PRC analysis, the slope of experimental PRCs at 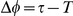 becomes steeper as the up-regulation of transcription increases due to greater light intensity or due to a larger chemical stimulus. If the slope at 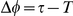 is less than −2, entrained oscillations are destabilized due to the occurrence of period-doubling bifurcations. The present results as well as those by Gonze and Goldbeter revealed that period-2, period-4, or chaotic oscillations occur due to the sequence of period-doubling bifurcations [28]. To our knowledge, however, oscillations with higher-order periods or chaos have not been observed experimentally due to very high light intensity. One possible explanation is that such complex oscillations are extremely difficult to measure even if the data themselves are chaotic because their time-series would be very similar to stable oscillation with intrinsic and/or external noise. Another explanation, which present results may provide, is that the light adaptation mechanism may prevent the occurrence of these complex oscillations to suppress the occurrence of period-doubling bifurcations. Heintzen et al. revealed that in *Neurospora*, *vvd* genes underlie light adaptation in *frq* induction [37], [57]. We predict that the dysfunction of light adaptation to lose *vvd* may cause the entrained oscillation of FRQ to destabilize in *Neurospora*. In mammal, there is no direct experimental evidence that up-regulation of clock genes by light show light adaptation. We herein estimated the transcriptional response for the m*per*1 gene during light period to be transient (black line, lower panel in Figure 1C). In contrast, the estimated transcriptional response for the m*per*2 gene increases very slowly during light period (purple line, lower panel in Figure 1C). Computational results show that up-regulation followed by adaptation to light increases entrainability under LD cycles while slow response to light hardly affects entrainability. Therefore, present results suggest that transient response of m*Per*1 gene to light might be critical for the stable entrainment.

In this study, we assumed that light adaptation is complete by the light-dark transition at the latest. However, it is possible that the basal level of gene expression is not reached until after the light-dark transition into the dark period. Present results shown in Figure 4A for example, imply that length of the period for the completion of adaptation enhances the entrainment. Therefore, we expect that circadian oscillations are more likely to be entrained by LD cycles if adaptation is not complete by the light-dark transition and it is not abrupt.

The length of photoperiod also affects the entrainment. Numerical results presented in Figures 4 and 5 generally show that entrainability under 24-h LD cycles increases as the duration of transcriptional response to light increases. If the duration of transcriptional response to light can be corresponded to the duration of photoperiod, we can expect that entrainability increases as the duration of photoperiod increases. Moreover, present results suggest that the magnitude of the enhancement of entrainment caused by the elongation of photoperiod, can be largely affected by whether elongation of photoperiod increases the period for maximum transcriptional response to light (*T*_s_), or increases the period for suppression of light induction (*T*_d_). In fact, parameter region for the entrainment becomes more than twice if elongation of photoperiod increases the period for suppression of light induction and then, the period for suppression of light induction was increased from 6 h to 9 h for example, corresponding to that the duration of photoperiod was increased by 3 h (Figure 4A). Thus, these results may raise the possibility that short photoperiod can cause the low entrainability as a result of the decrease for the period of light adaptation.

Recently many realistic models on circadian rhythms have been proposed in which numerous genes and proteins are involved [23]–[27], [29], [30]. The results from a simple model studied in the present article may not be applicable directly to more complex model, realistic models. We additionally examined the effect of the light adaptation on the region of entrainment region in the model published previously by Leloup and Goldbeter [23], [24] with three sets of model parameters (Figure S4). For one of three parameter sets (Figure S4A), the region of entrainment does not monotonically enlarge as duration of maximum transcriptional response shortens which is not consistent with our results. This suggests that the predictions derived from very simple models might not be always applicable to a broader class of models with many structures and numerous reaction steps. However, for two of three parameter sets in that model (Figure S4B and S4C), region of entrainment enlarges as duration of maximum transcriptional response shortens which is consistent with our results. For these two parameter conditions, the expression rate of *Reverb α* gene is fixed as 0 and then the structure of gene-protein network becomes simpler, that may cause the enlargement of domain of entrainment by suppression of transcription followed by light induction.

For the simplicity of argument, we used a simple model to predict basic principles for the entrainment of circadian rhythms. However, circadian rhythms also occur at multi-cellular level [1], [6]. Certainly, it is possible that synchronization or orchestration between cells and organs affect the entrainment of circadian oscillations by environmental cycles and such a case, our predictions above obtained by a single cell model cannot be applied to the behavior level response. We wish to study the generality of our conclusions in the future.

## Methods

### Estimation of time courses of the transcription rate *v_s_*

First, by adding Eqs. 2 and 3, we can derive an equation corresponding to the temporal change of the total protein, 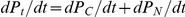. We substituted the experimental data of mRNA and protein under DD condition [44], [45] or LD cycles [10], [18] into *M* and *P_t_* of the equation, respectively. Then time-series of the concentration of protein in the cytosol, *P_C_*, that most perfectly fitted the experimental data was estimated under the condition that *P_C_* does not exceed *P_t_*. Subsequently, we substituted the experimental data of mRNA and the *P_N_* (i.e. *P_N_ = P_t_−P_C_*) into Eq. 1, and then estimated time-series of transcription rate *v_s_* using the parameters except for *v_s_* which are estimated from DD data [44], [45].

### Numerical methods

To numerically integrate Eqs. 1–3 to obtain the time evolution of mRNA (*M*) and proteins (*P_C_*) and (*P_N_*), we used the fourth-order Runge-Kutta method with double precision numbers.

We also analyzed the bifurcations of periodic oscillations to find changes in the stability of periodic oscillations (Figure S5) and the emergence of complex oscillations for choosing parameters [50], [58]. Bifurcations occur when the stability of periodic oscillations changes by varying system parameters [59]–[61]. To investigate these bifurcations, we used a method involving a stroboscopic map, also called the Poincaré map [59]–[61]. Thereby, the analysis of a periodic oscillation is reduced to that of a fixed point on the Poincaré map. In Eqs. 1–3, a parameter that represents the effect of LD cycles, however, changes discontinuously with time. Therefore, we modified the method that has been proposed to investigate the bifurcations of periodic oscillations observed in a discontinuous dynamical system [61]. A detailed description of the numerical method is provided in the Text S1.

## Supporting Information

Figure S1 **Desynchronous oscillations in the form of a quasi-periodic (A) and a chaotic oscillation (B).** In left figures of each panel, the solid and dashed lines in the upper figure correspond to the time courses for concentrations of mRNA, *M*, in cytosol and of protein, *P_N_*, in the nucleus. The solid green line indicates the time course of mRNA production. The solid purple line in the lower panel represents temporal variations in the transcription rate under 12 h∶12 h LD cycles. The right figures in each panel indicate the phase portraits projected onto the (*M*, *P_N_*)-plane. In the phase portraits, the solid line and filled circles represent the orbits that are obtained by projecting the trajectory onto the (*M*, *P_N_* )-plane and the iterated point every 24 h, i.e. the points on the Poincaré map. In both cases, duration of maximum transcriptional response *T_s_* and decay-time *T_d_* are fixed as 6 h. The maximum value of the transcriptional responses, *X^max^* is 0.125 (A) and 3.375 (B). Parameters except for *T_s_*, *T_d_*, *X^max^* are the same as in Figure 3. The LD cycles are represented by the white and black bars, respectively.
(TIF)

Figure S2 **Effects of light adaptation on entrainment of circadian oscillations with a period of 25 h.** We observed dynamic behavior as a function of the maximum response (*X^max^*) and duration of maximum response (*T_s_*) when up-regulation of transcription by light is in the form of light adaptation (A) and slow response (B). The lower (open circles) and upper (closed circles) limits correspond to the saddle-node and the period-doubling bifurcation points. Gray shading in A and B indicates condition for the circadian oscillations entrained by 12 h∶12 h LD cycles. *T_s_*+*T_d_* and *T_s_*+*T_r_* were fixed as 12 h. The insets in A and B are enlarged diagrams in a certain range of duration *T_s_*. Parameter except for *v_s_* are the same as in Figure 3. *v_s_* = 2.5 nM/h.
(TIF)

Figure S3 **A period-4 oscillation when the transcriptional response is of square-wave.** In the left figure, mRNA (solid line), protein (dashed line), mRNA production (solid green line), and temporal variations in the transcription rate (purple line) under 24-h LD cycles are shown. The right figure is phase portrait with points on the Poincaré map in which solid line corresponds to the projected trajectory on the (*M*, *P_N_*)-plane and filled circles are the iterated points every 24 h. The maximum value of the transcriptional responses *X^max^* was fixed as 0.75. Parameters except for *X^max^* are the same as in Figure 3.
(TIF)

Figure S4 **Effect of light adaptation on entrainment for detailed mammalian circadian clock models** [**23]**, [24****] **.** We observed dynamic behavior as a function of the maximum response and duration of maximum response *T_s_* for (A) the model incorporating a negative regulator, *REV-ERB α* [23], (B) the model without *REV-ERB α*, and (C) the model without *REV-ERB α* and the negative autoregulation by BMAL1 [24]. The entrainment range consists of lower and upper limits of the highest values in transcriptional response, *X^max^*, at which the circadian oscillation entrained by LD cycles can be observed in cases where temporal variation of the transcriptional response to light varies in light adaptation. The open symbols indicate the saddle-node (*SN*) bifurcation points. The closed squares and circles correspond to the period-doubling (*P_d_*) and the Neimark-Sacker (*NS*) bifurcation points, respectively. The insets in the panel *A* and *B* are enlarged diagrams in a certain range of duration *T_s_*. Parameter values are the same as in [23] for (A), are the same as in the parameter sets 1 and 3 represented in [24] for (B) and (C), respectively.
(TIF)

Figure S5 **Typical examples of two-parameter bifurcation diagram of a periodic oscillation.** We observed dynamic behavior as a function of the maximum transcriptional response (*X^max^*) and period of LD cycles (*T*) when up-regulation of transcription by light is in the form of light adaptation (A) and slow response (B). The right panels in Aii and Bii are enlarged diagrams in a certain range of *X^max^* in Ai and Bi. Gray shading indicates condition for the circadian oscillations entrained by light-dark cycles with the forcing period, *T*. The solid, dashed, and dotted lines indicate saddle-node bifurcation (*SN*), period-doubling bifurcation (*P_d_*), and Neimark-Sacker bifurcation (*NS*) sets. The duration of *T_s_* = 3*T*/24 h (A, B), *T_d_* = 9*T*/24 (A), and *T_r_* = 9*T*/24 (B). Parameters except for *T_s_*, *T_d_*, and *T_r_* in Eqs. 1–3 are the same as in Figure 3.
(TIF)

Text S1 **Supporting Material.**
(PDF)
