# Supplementary figures and images for: Modeling Light Adaptation in Circadian Clock: Prediction of the Response That Stabilizes Entrainment
Source: PLoS One. 2011 Jun 16;6(6):e20880. doi: 10.1371/journal.pone.0020880 (PMC3116846; doi:10.1371/journal.pone.0020880)

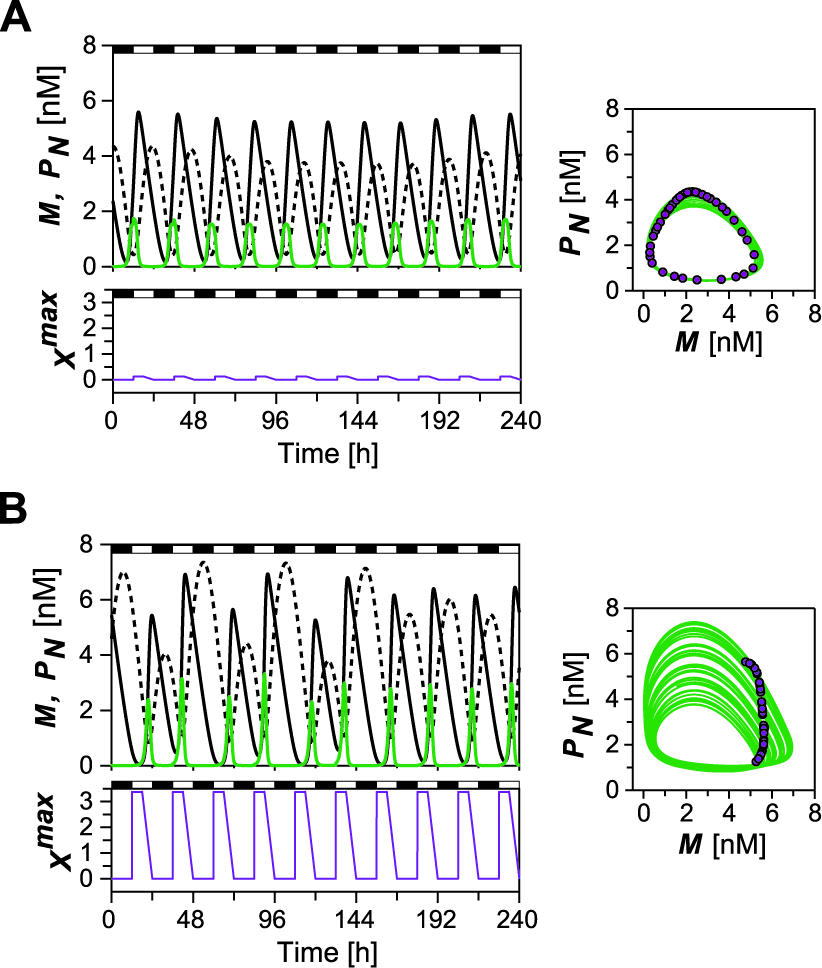

Supplement: Figure S1 — Desynchronous oscillations in the form of a quasi-periodic (A) and a chaotic oscillation (B). In left figures of each panel, the solid and dashed lines in the upper figure correspond to the time courses for concentrations of mRNA, M, in cytosol and of protein, PN, in the nucleus. The solid green line indicates the time course of mRNA production. The solid purple line in the lower panel represents temporal variations in the transcription rate under 12 h∶12 h LD cycles. The right figures in each panel indicate the phase portraits projected onto the (M, PN)-plane. In the phase portraits, the solid line and filled circles represent the orbits that are obtained by projecting the trajectory onto the (M, PN )-plane and the iterated point every 24 h, i.e. the points on the Poincaré map. In both cases, duration of maximum transcriptional response Ts and decay-time Td are fixed as 6 h. The maximum value of the transcriptional responses, Xmax is 0.125 (A) and 3.375 (B). Parameters except for Ts, Td, Xmax are the same as in Figure 3. The LD cycles are represented by the white and black bars, respectively. (TIF) [file pone.0020880.s001.tif]

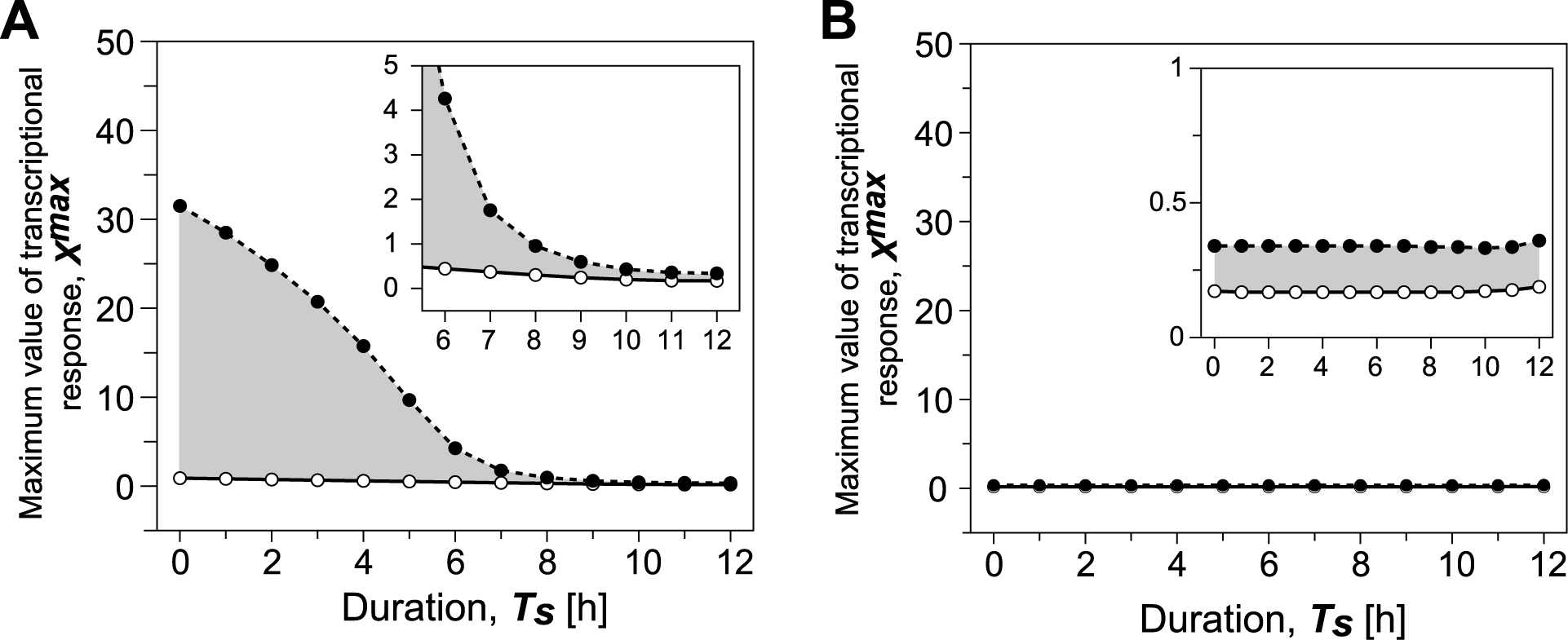

Supplement: Figure S2 — Effects of light adaptation on entrainment of circadian oscillations with a period of 25 h. We observed dynamic behavior as a function of the maximum response (Xmax) and duration of maximum response (Ts) when up-regulation of transcription by light is in the form of light adaptation (A) and slow response (B). The lower (open circles) and upper (closed circles) limits correspond to the saddle-node and the period-doubling bifurcation points. Gray shading in A and B indicates condition for the circadian oscillations entrained by 12 h∶12 h LD cycles. Ts+Td and Ts+Tr were fixed as 12 h. The insets in A and B are enlarged diagrams in a certain range of duration Ts. Parameter except for vs are the same as in Figure 3. vs = 2.5 nM/h. (TIF) [file pone.0020880.s002.tif]

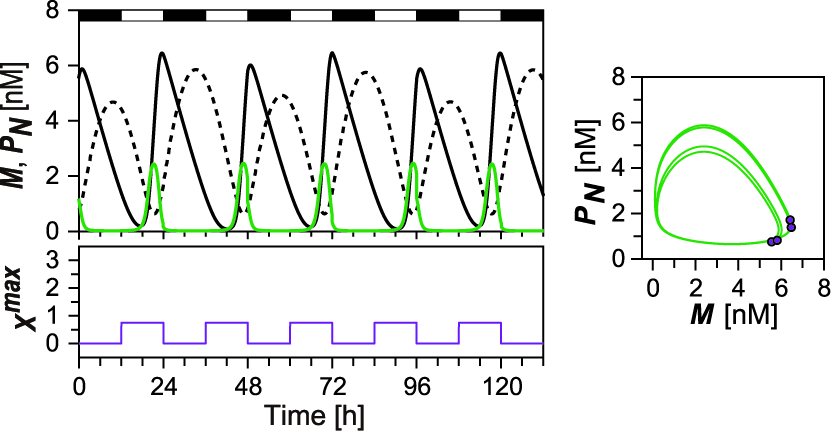

Supplement: Figure S3 — A period-4 oscillation when the transcriptional response is of square-wave. In the left figure, mRNA (solid line), protein (dashed line), mRNA production (solid green line), and temporal variations in the transcription rate (purple line) under 24-h LD cycles are shown. The right figure is phase portrait with points on the Poincaré map in which solid line corresponds to the projected trajectory on the (M, PN)-plane and filled circles are the iterated points every 24 h. The maximum value of the transcriptional responses Xmax was fixed as 0.75. Parameters except for Xmax are the same as in Figure 3. (TIF) [file pone.0020880.s003.tif]

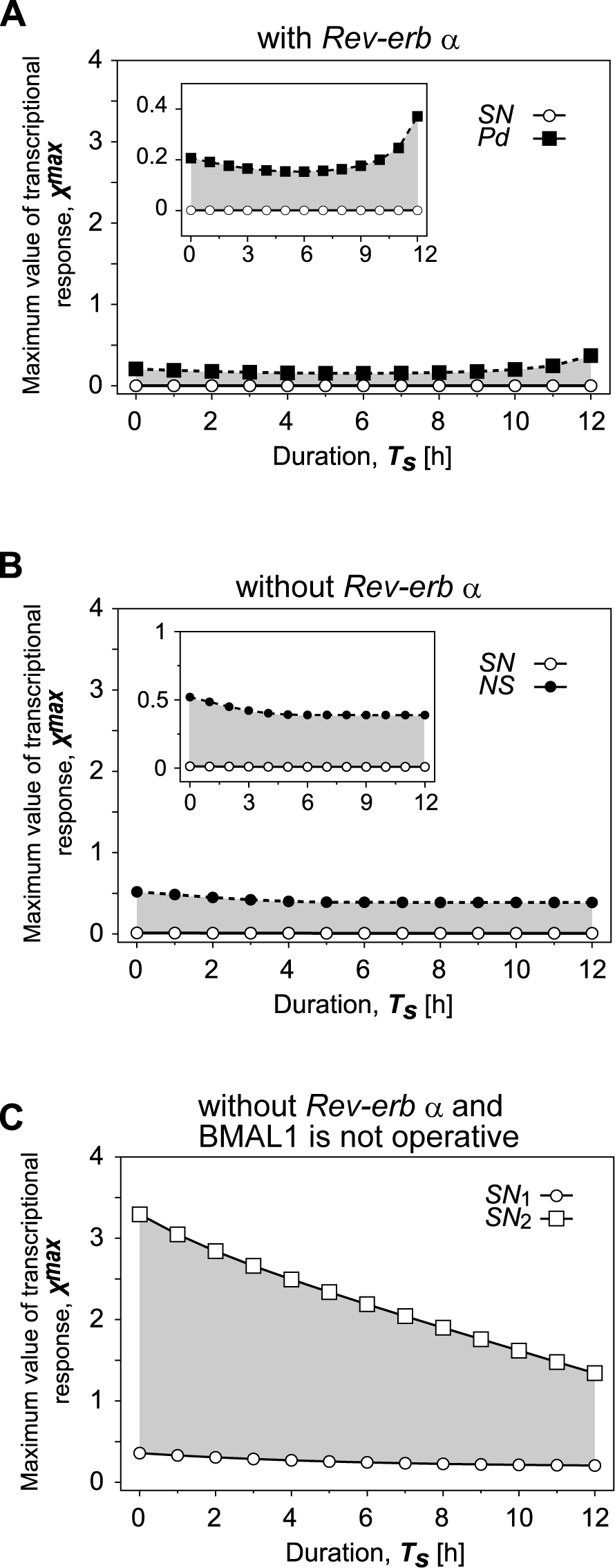

Supplement: Figure S4 — Effect of light adaptation on entrainment for detailed mammalian circadian clock models [23] , [24] . We observed dynamic behavior as a function of the maximum response and duration of maximum response Ts for (A) the model incorporating a negative regulator, REV-ERB α [23], (B) the model without REV-ERB α, and (C) the model without REV-ERB α and the negative autoregulation by BMAL1 [24]. The entrainment range consists of lower and upper limits of the highest values in transcriptional response, Xmax, at which the circadian oscillation entrained by LD cycles can be observed in cases where temporal variation of the transcriptional response to light varies in light adaptation. The open symbols indicate the saddle-node (SN) bifurcation points. The closed squares and circles correspond to the period-doubling (Pd) and the Neimark-Sacker (NS) bifurcation points, respectively. The insets in the panel A and B are enlarged diagrams in a certain range of duration Ts. Parameter values are the same as in [23] for (A), are the same as in the parameter sets 1 and 3 represented in [24] for (B) and (C), respectively. (TIF) [file pone.0020880.s004.tif]

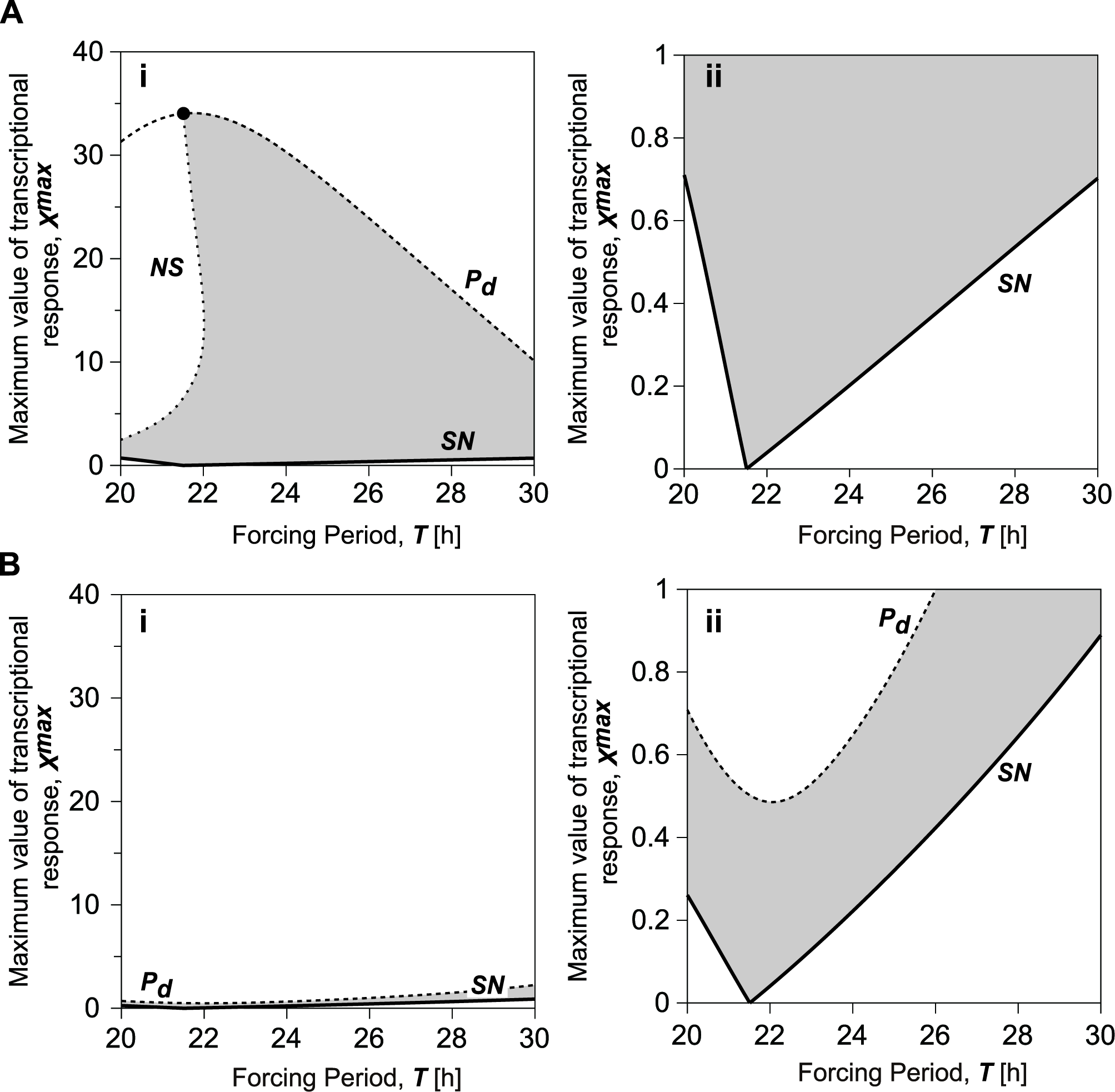

Supplement: Figure S5 — Typical examples of two-parameter bifurcation diagram of a periodic oscillation. We observed dynamic behavior as a function of the maximum transcriptional response (Xmax) and period of LD cycles (T) when up-regulation of transcription by light is in the form of light adaptation (A) and slow response (B). The right panels in Aii and Bii are enlarged diagrams in a certain range of Xmax in Ai and Bi. Gray shading indicates condition for the circadian oscillations entrained by light-dark cycles with the forcing period, T. The solid, dashed, and dotted lines indicate saddle-node bifurcation (SN), period-doubling bifurcation (Pd), and Neimark-Sacker bifurcation (NS) sets. The duration of Ts = 3T/24 h (A, B), Td = 9T/24 (A), and Tr = 9T/24 (B). Parameters except for Ts, Td, and Tr in Eqs. 1–3 are the same as in Figure 3. (TIF) [file pone.0020880.s005.tif]
