# Supplementary material for: Modeling Light Adaptation in Circadian Clock: Prediction of the Response That Stabilizes Entrainment
Source: PLoS One. 2011 Jun 16;6(6):e20880. doi: 10.1371/journal.pone.0020880 (PMC3116846; doi:10.1371/journal.pone.0020880)
Supplement: Text S1 — Supporting Material. (PDF) [file pone.0020880.s006.pdf]

Supporting Material

Text S1

Modeling Light Adaptation in Circadian Clock: Prediction of the Response that Stabilizes Entrainment

Kunichika Tsumoto, Gen Kurosawa, Tetsuya Yoshinaga, and Kazuyuki Aihara

Detailed Method for Numerical Bifurcation Analyses

A composite dynamical system

In our model, an autonomous circadian oscillator shown in Eqs. 1-3 is driven by light-dark (LD) cycles. We hypothetically consider three types of the transcriptional response, X, during light phase (see Methods). The temporal changes in $X(t)$ for the transcriptional response with light adaptation (Figure 2A) and with slow response (Figure 2B) are expressed as

$$X(t)=\begin{cases} X^{max} & (0 \le t < T_s) \\ \dfrac{X^{max}(T_s+T_d-t)}{T_d} & (T_s \le t < T_s+T_d), \\ 0 & (T_s+T_d \le t < T) \end{cases} \tag{S1}$$

and

$$X(t)=\begin{cases} \dfrac{X^{max}}{T_r}t & (0 \le t < T_r) \\ X^{max} & (T_r \le t < T_s+T_r), \\ 0 & (T_s+T_r \le t < T) \end{cases} \tag{S2}$$

where T, T_s, T_d, and T_r correspond to the period of the LD cycle, the duration that the transcriptional response is sustained at the maximum value, X^{max}, the decay-time, and the rise-time. The first and second equations in Eq. S1 or S2 correspond to the temporal variations in the transcriptional response during the light phase of the LD cycle, while the third equation represents the change during the dark phase. Then, the circadian oscillator during the dark phase and during the light phase behaves the same as the autonomous system in Eqs. 1-3 with constant parameters v_s and $v_s(1+X^{max})$. However, when the transcriptional response varies with time during the decay-time or the rise-time the circadian oscillator behaves as a non-autonomous system whose the parameter X varies over time. Therefore, the system in Eqs. 1-3 with the light adaptation or slow response in the transcriptional response under the LD cycles can be formalized as a composite dynamical system so that the two corresponding autonomous systems and a unique non-autonomous one are successively switched over time.

Poincaré map of composite dynamical system

Bifurcations occur when the stability of periodic oscillations changes by varying system parameters. To investigate these bifurcations, we use a method involving a stroboscopic map, also called the Poincaré map [50,59,60]. Thereby, the analysis of a

periodic oscillation is reduced to that of a fixed point on the Poincaré map.

Only the non-autonomous system with the temporal variation of the transcriptional response given by Eq. S1 is considered in the following. The same procedure can be applied by replacing the descriptions in regard to the first and second equations in Eq. S1 with those in regard to the second and the first equations in Eq. S2. Now let us consider the following non-autonomous differential equations consisting of Eqs. 1-3 and a periodic parameter variation of either Eq. S1 or S2 during the time satisfying $t - t_0 (\mathrm{mod} T) \in [0, T)$:

$$\frac{dx}{dt} = f(t, x, \lambda) = \begin{cases} f_1(x, \lambda_0, \lambda_a) & (t_0 \le t < t_0 + T_s) \\ f_2(t, x, \lambda_0, \lambda_a, \lambda_b, \lambda_c) & (t_0 + T_s \le t < t_0 + T_s + T_d), \\ f_3(x, \lambda_0) & (t_0 + T_s + T_d \le t < t_0 + T) \end{cases} \tag{S3}$$

where $t \in R$ denotes the time, x is the state vector $x = (q^1, q^2, q^3)^T$,where $(\ )^T$ represents the transpose operation, $\lambda_0 \in R^{m-3}$ denotes common parameters for f, and $\lambda_a \in R$ is a parameter specifying f_1 and f_2, whereas $\lambda_b, \lambda_c \in R$ are parameters specifying f_2. The parameters λ_a, λ_b, and λ_c correspond to X^{max}, T_s, and T_d. We also assume that the function, f, in Eq. S3 is periodic over time with the period of the LD cycle, T, i.e., $f(t + T, x, \lambda) = f(t, x, \lambda)$ for all t. Assume that the whole solution to Eq. S3 is described as a mixed solution of the first, second, and third equations of Eq. S3. Then, the solution with initial condition $x = x_0$ at $t = t_0$ is represented by

$$x(t) = \varphi(t, \lambda; t_0, x_0) = \varphi(t, \lambda_0, \lambda_a, \lambda_b, \lambda_c; t_0, x_0). \tag{S4}$$

Let φ_1, φ_2, and φ_3 correspond to solutions to the first, second, and third equations of Eq. S3

$$x_1(t) = \varphi_1(t, \lambda_0, \lambda_a; t_0, x_0), \ (t_0 \le t < t_0 + T_s),$$

$$x_2(t) = \varphi_2(t, \lambda_0, \lambda_a, \lambda_b, \lambda_c; t_0 + T_s, \varphi_1(t_0 + T_s, \lambda_0, \lambda_a; t_0, x_0)), \\ (t_0 + T_s \le t < t_0 + T_s + T_d)$$

and

$$x_3(t) = \varphi_3(t, \lambda_0; t_0 + T_s + T_d, \varphi_2(t_0 + T_s + T_d, \lambda_0, \lambda_a, \lambda_b, \lambda_c; \\ t_0 + T_s, \varphi_1(t_0 + T_s, \lambda_0, \lambda_a; t_0, x_0))), (t_0 + T_s + T_d \le t < t_0 + T).$$

Then, the Poincaré map

$$\begin{aligned} S_T &: R^n \to R^n \\ x_0 &\mapsto S_T(x_0) = \varphi(t_0 + T, \lambda_0, \lambda_a, \lambda_b, \lambda_c; t_0, x_0) \end{aligned} \tag{S5}$$

is defined as a composite map, $S_T = S_{T3} \circ S_{T2} \circ S_{T1}$, to avoid discontinuity in the derivative of the solution at $t = t_0$, $t = t_0 + T_s$, and $t = t_0 + T_s + T_d$, where S_{T1}, S_{T2}, and S_{T3} are given by the submaps:

$$S_{T1}: R^n \to R^n$$
$$x_0 \mapsto x_1 = \varphi_1(t_0 + T_s, \lambda_0, \lambda_a; t_0, x_0),$$
$$S_{T2}: R^n \to R^n$$
$$x_1 \mapsto x_2 = \varphi_2(t_0 + T_s + T_d, \lambda_0, \lambda_a, \lambda_b, \lambda_c; t_0 + T_s, x_1),$$

and

$$S_{T3}: R^n \to R^n$$
$$x_2 \mapsto x_3 = \varphi_3(t_0 + T, \lambda_0; t_0 + T_s + T_d, x_2).$$

In this way, the behavior of a periodic oscillation can be reduced to that of a fixed point on the Poincaré map.

The numerical calculation of the fixed point

Considering the fixed point on the Poincaré map, we define a fixed-point equation as

$$F(x_0) := x_0 - S_T(x_0) = 0, \tag{S6}$$

where $x_0 \in R^n$ denotes the initial value at $t = t_0$. Since the fixed point equation of Eq. S6 cannot be solved analytically, we use a numerical approach to computing it such as Newton's method. The recurrent formula for Newton's method is given by

$$\begin{cases} v^{(k+1)} = v^{(k)} + \delta \\ DF\left(v^{(k)}\right)\delta + F\left(v^{(k)}\right) = 0 \end{cases}, \quad k = 0, 1, 2, \cdots,$$

where v is an unknown variable, δ is the correction term, and DF is the Jacobian matrix of F denoted by

$$DF = \left[I_n - \frac{\partial S_T(x_0)}{\partial x_0} \right],$$

where I_n is the $n \times n$ identity matrix. Consequently, we need to differentiate the Poincaré map, S_T, with respect to the initial value, x_0, to obtain each element of the Jacobian matrix. Then, the derivative of the S_T with regard to x_0 is expressed by

$$\frac{\partial S_T}{\partial x_0}(x_0) = \frac{\partial \varphi}{\partial x_0}(t_0 + T, \lambda_0, \lambda_a, \lambda_b, \lambda_c; t_0, x_0).$$

The derivatives, $\partial\varphi/\partial x_0$, however, cannot be directly defined due to discontinuities in the derivative of the solution. To avoid the impossibility of that derivative at $t = t_0 + T_s$, $t_0 + T_s + T_d$, and $t_0 + T$, the first derivative of the S_T with respect to initial value x_0 is given by obtaining the derivatives of the submaps, successively, i.e.,

$$\frac{\partial S_T(x_0)}{\partial x_0} = \frac{\partial S_{T3}(x_2)}{\partial x_2}\frac{\partial S_{T2}(x_1)}{\partial x_1}\frac{\partial S_{T1}(x_0)}{\partial x_0}$$

$$= \frac{\partial \varphi_3}{\partial x_2}(t_0 + T, \lambda_0; t_0 + T_s + T_d, x_2)$$

$$\times \frac{\partial \varphi_2}{\partial x_1}(t_0 + T_s + T_d, \lambda_0, \lambda_a, \lambda_b \lambda_c; t_0 + T_s, x_1) \tag{S7}$$

$$\times \frac{\partial \varphi_1}{\partial x_0}(t_0 + T_s, \lambda_0, \lambda_a; t_0, x_0).$$

In Eq. S7, the derivatives of φ_i, for i = 1, 2, 3, regard to initial value x_k for k = 0, 1, 2, which correspond to fundamental matrix solutions, i.e., $\partial\varphi_i / \partial x_k$, can be obtained by solving each of the first-order variational equations:

$$\frac{d}{dt}\left(\frac{\partial \varphi_1}{\partial x_0}\right) = \frac{\partial f_1}{\partial x}\frac{\partial \varphi_1}{\partial x_0} \quad \text{with} \quad \left.\frac{\partial \varphi_1}{\partial x_0}\right|_{t=t_0} = I, \tag{S8}$$

$$\frac{d}{dt}\left(\frac{\partial \varphi_2}{\partial x_1}\right) = \frac{\partial f_2}{\partial x}\frac{\partial \varphi_2}{\partial x_1} \quad \text{with} \quad \left.\frac{\partial \varphi_2}{\partial x_1}\right|_{t=t_0+T_s} = I, \tag{S9}$$

and

$$\frac{d}{dt}\left(\frac{\partial \varphi_3}{\partial x_2}\right) = \frac{\partial f_3}{\partial x}\frac{\partial \varphi_3}{\partial x_2} \quad \text{with} \quad \left.\frac{\partial \varphi_3}{\partial x_2}\right|_{t=t_0+T_s+T_d} = I, \tag{S10}$$

and putting $t = t_0 + T_s$, $t_0 + T_s + T_d$, and $t_0 + T$ in the respective solutions to Eqs. S8–S10.

The method for the bifurcation analysis

Next, we summarize a method of calculating a bifurcation set on an arbitrary two-parameter plane. The numerical determination of a bifurcation set is accomplished by using the method proposed by Kawakami [60] so that the accurate location of a fixed point and a bifurcation parameter value are calculated by solving the fixed point equation and the bifurcation condition simultaneously.

First, we select $\lambda_1 \in \lambda$ as a bifurcation parameter and $\lambda_2 \in \lambda$, except for λ_1, as a control parameter and also assume that a certain class of bifurcations occurs at the value of λ_1^* in the situation where all the other parameters are fixed. The codimension-one bifurcations that the circadian system could produce are saddle-node, period-doubling, and Neimark-Sacker bifurcations [50,58]. The conditions for the three bifurcations correspond to the critical distribution of the eigenvalue in the characteristic equation for

Eq. S6: $\mu = +1, \mu = -1$, and $|\mu| = 1$, respectively [50,58,59]. Then, let us consider the following simultaneous equation consisting of the fixed point equation of Eq. S6 and the characteristic equation for Eq. S6:

$$F_{set} := \begin{bmatrix} x_0 - S_T(x_0) \\ \det(\mu^* I_n - D S_T(x_0^*)) \end{bmatrix} = 0, \tag{S11}$$

where $D S_T(x_0^*)$ denotes the derivative of the Poincaré map, S_T, i.e., $D S_T(x_0^*) = \partial S_T(x_0) / \partial x_0 \big|_{x_0 = x_0^*}$ and μ^* represent the value corresponding to the bifurcation condition, e.g., this is $\mu^* = 1$ for calculating a saddle-node bifurcation set. Equation S11 can be solved for the unknown variables, x_0 and λ_1, by using Newton's method. The Jacobian matrix of F_{set} required by Newton's method is

$$DF_{set} = \begin{bmatrix} I_n - \dfrac{\partial S_T(x_0)}{\partial x_0} & -\dfrac{\partial S_T(x_0)}{\partial \lambda_1} \\ \dfrac{\partial \chi(\mu^*)}{\partial x_0} & \dfrac{\partial \chi(\mu^*)}{\partial \lambda_1} \end{bmatrix}, \tag{S12}$$

where $\chi(\mu^*)$ denotes the characteristic equation which is given as a determinant of the following $n \times n$ matrix:

$$P(x_0) := \mu^* I_n - \frac{\partial S_T(x_0)}{\partial x_0}, \tag{S13}$$

i.e., $\chi(\mu^*)$:= det (P) = 0. In Eq. S12, the derivatives of the characteristic equation, $\chi(\mu^*)$, with respect to initial value x_0 can be obtained by using

$$\frac{\partial \chi(\mu^*)}{\partial x_0} = \sum_{i=1}^{n} \det(P_i), \tag{S14}$$

where P_i represents matrices that are differentiated from each element of the ith column of P with regard to x_0. The derivatives of the characteristic equation related to parameter λ_1 are the same as that in Eq. S14. Consequently, for calculating each element of the Jacobian matrix of Eq. S12, it is necessary to obtain the first and second derivatives of Poincaré map S_T with respect to the initial condition and the system parameter:

$$\frac{\partial S_T}{\partial \lambda_1} = \frac{\partial \varphi}{\partial \lambda_1}, \frac{\partial}{\partial q_0^j}\left(\frac{\partial S_T}{\partial x_0}\right) = \frac{\partial}{\partial q_0^j}\left(\frac{\partial \varphi}{\partial x_0}\right), \text{ and } \frac{\partial}{\partial \lambda_1}\left(\frac{\partial S_T}{\partial x_0}\right) = \frac{\partial}{\partial \lambda_1}\left(\frac{\partial \varphi}{\partial x_0}\right), \tag{S15}$$

for j=1, 2, 3, where q_0^j denotes an element in the initial value x_0. Then, each derivative in Eq. S15 as well as the first derivative of Poincaré map S_T with regard to the initial

value, x_0, can be given by obtaining the derivatives of the submaps, successively, i.e.,

$$\frac{\partial\varphi}{\partial\lambda_1}=\frac{\partial\varphi_3}{\partial\lambda_1}+\frac{\partial\varphi_3}{\partial x_2}\frac{\partial\varphi_2}{\partial\lambda_1}+\frac{\partial\varphi_3}{\partial x_2}\frac{\partial\varphi_2}{\partial x_1}\frac{\partial\varphi_1}{\partial\lambda_1}, \tag{S16}$$

$$\begin{aligned}\frac{\partial}{\partial q_0^j}\left(\frac{\partial\varphi}{\partial x_0}\right)&=\frac{\partial}{\partial q_0^j}\left(\frac{\partial\varphi_3}{\partial x_2}\right)\frac{\partial\varphi_2}{\partial x_1}\frac{\partial\varphi_1}{\partial x_0}\\&+\frac{\partial\varphi_3}{\partial x_2}\frac{\partial}{\partial q_0^j}\left(\frac{\partial\varphi_2}{\partial x_2}\right)\frac{\partial\varphi_1}{\partial x_0}+\frac{\partial\varphi_3}{\partial x_2}\frac{\partial\varphi_2}{\partial x_1}\frac{\partial}{\partial q_0^j}\left(\frac{\partial\varphi_1}{\partial x_2}\right).\end{aligned} \tag{S17}$$

and

$$\begin{aligned}\frac{\partial}{\partial\lambda_1}\left(\frac{\partial\varphi}{\partial x_0}\right)&=\frac{\partial}{\partial\lambda_1}\left(\frac{\partial\varphi_3}{\partial x_2}\right)\frac{\partial\varphi_2}{\partial x_1}\frac{\partial\varphi_1}{\partial x_0}\\&+\frac{\partial\varphi_3}{\partial x_2}\frac{\partial}{\partial\lambda_1}\left(\frac{\partial\varphi_2}{\partial x_1}\right)\frac{\partial\varphi_1}{\partial x_0}+\frac{\partial\varphi_3}{\partial x_2}\frac{\partial\varphi_2}{\partial x_1}\frac{\partial}{\partial\lambda_1}\left(\frac{\partial\varphi_1}{\partial x_0}\right),\end{aligned} \tag{S18}$$

In particular, note that the derivatives of the Poincaré map with respect to parameters specifying f_i, for i = 1, 2, in Eq. S3, i.e., λ_a, λ_b, and λ_c are

$$\frac{\partial\varphi}{\partial\lambda_a}=\frac{\partial\varphi_3}{\partial x_2}\frac{\partial\varphi_2}{\partial\lambda_a}+\frac{\partial\varphi_3}{\partial x_2}\frac{\partial\varphi_2}{\partial x_1}\frac{\partial\varphi_1}{\partial\lambda_a}, \tag{S19}$$

$$\frac{\partial\varphi}{\partial\lambda_b}=\frac{\partial\varphi_3}{\partial\lambda_b}+\frac{\partial\varphi_3}{\partial x_2}\frac{\partial\varphi_2}{\partial\lambda_b}, \tag{S20}$$

$$\frac{\partial\varphi}{\partial\lambda_c}=\frac{\partial\varphi_3}{\partial x_2}\frac{\partial\varphi_2}{\partial\lambda_c}, \tag{S21}$$

$$\begin{aligned}\frac{\partial}{\partial\lambda_a}\left(\frac{\partial\varphi}{\partial x_0}\right)&=\frac{\partial}{\partial\lambda_a}\left(\frac{\partial\varphi_3}{\partial x_2}\right)\frac{\partial\varphi_2}{\partial x_1}\frac{\partial\varphi_1}{\partial x_0}\\&+\frac{\partial\varphi_3}{\partial x_2}\frac{\partial}{\partial\lambda_a}\left(\frac{\partial\varphi_2}{\partial x_1}\right)\frac{\partial\varphi_1}{\partial x_0}+\frac{\partial\varphi_3}{\partial x_2}\frac{\partial\varphi_2}{\partial x_1}\frac{\partial}{\partial\lambda_a}\left(\frac{\partial\varphi_1}{\partial x_0}\right),\end{aligned} \tag{S22}$$

$$\begin{aligned}\frac{\partial}{\partial\lambda_b}\left(\frac{\partial\varphi}{\partial x_0}\right)&=\frac{\partial}{\partial\lambda_b}\left(\frac{\partial\varphi_3}{\partial x_2}\right)\frac{\partial\varphi_2}{\partial x_1}\frac{\partial\varphi_1}{\partial x_0}\\&+\frac{\partial\varphi_3}{\partial x_2}\frac{\partial}{\partial\lambda_b}\left(\frac{\partial\varphi_2}{\partial x_1}\right)\frac{\partial\varphi_1}{\partial x_0}+\frac{\partial\varphi_3}{\partial x_2}\frac{\partial\varphi_2}{\partial x_1}\frac{\partial}{\partial\lambda_b}\left(\frac{\partial\varphi_1}{\partial x_0}\right),\end{aligned} \tag{S23}$$

$$\frac{\partial}{\partial \lambda_c}\left(\frac{\partial \varphi}{\partial x_0}\right) = \frac{\partial}{\partial \lambda_c}\left(\frac{\partial \varphi_3}{\partial x_2}\right)\frac{\partial \varphi_2}{\partial x_1}\frac{\partial \varphi_1}{\partial x_0} + \frac{\partial \varphi_3}{\partial x_2}\frac{\partial}{\partial \lambda_c}\left(\frac{\partial \varphi_2}{\partial x_1}\right)\frac{\partial \varphi_1}{\partial x_0} + \frac{\partial \varphi_3}{\partial x_2}\frac{\partial \varphi_2}{\partial x_1}\frac{\partial}{\partial \lambda_c}\left(\frac{\partial \varphi_1}{\partial x_0}\right). \tag{S24}$$

In Eqs. S16–S18, each of the derivatives $\partial \varphi_i / \partial \lambda_1, \partial / \partial q_k^j (\partial \varphi_i / \partial x_k)$, and $\partial / \partial \lambda_1 (\partial \varphi_i / \partial x_k)$, for i, j = 1, 2, 3, and k = 0, 1, 2, can be obtained by integrating the following first- and second-order variational equations at each interval of $t = t_0$ to $t = t_0 + T_s$ for f_1, $t = t_0 + T_s$ to $t = t_0 + T_s + T_d$ for f_2, and $t = t_0 + T_s + T_d$ to $t = t_0 + T$ for f_3, respectively:

$$\frac{d}{dt}\left(\frac{\partial \varphi_i}{\partial \lambda_1}\right) = \frac{\partial f_i}{\partial x_i}\frac{\partial \varphi_i}{\partial \lambda_1} + \frac{\partial f_i}{\partial \lambda_1},$$

$$\frac{d}{dt}\left\{\frac{\partial}{\partial q_k^j}\left(\frac{\partial \varphi_i}{\partial x_k}\right)\right\} = \frac{\partial f_i}{\partial x_i}\frac{\partial}{\partial q_k^j}\left(\frac{\partial \varphi_i}{\partial x_k}\right) + \frac{\partial}{\partial q_k^j}\left(\frac{\partial f_i}{\partial x_i}\right)\left(\frac{\partial \varphi_i}{\partial x_k}\right)^2,$$

$$\frac{d}{dt}\left\{\frac{\partial}{\partial \lambda_1}\left(\frac{\partial \varphi_i}{\partial x_k}\right)\right\} = \frac{\partial f_i}{\partial x_i}\frac{\partial}{\partial \lambda_1}\left(\frac{\partial \varphi_i}{\partial x_k}\right) + \frac{\partial}{\partial q_k^j}\left(\frac{\partial f_i}{\partial x_i}\right)\frac{\partial \varphi_i}{\partial x_k}\frac{\partial \varphi_i}{\partial \lambda_1} + \frac{\partial}{\partial \lambda_1}\left(\frac{\partial f_i}{\partial x_i}\right)\frac{\partial \varphi_i}{\partial x_k},$$

with

$$\left.\frac{\partial \varphi_1}{\partial \lambda_1}\right|_{t=t_0} = \left.\frac{\partial \varphi_2}{\partial \lambda_1}\right|_{t=t_0+T_s} = \left.\frac{\partial \varphi_3}{\partial \lambda_1}\right|_{t=t_0+T_s+T_d} = 0,$$

$$\left.\frac{\partial}{\partial q_k^j}\left(\frac{\partial \varphi_1}{\partial x_0}\right)\right|_{t=t_0} = \left.\frac{\partial}{\partial q_k^j}\left(\frac{\partial \varphi_2}{\partial x_1}\right)\right|_{t=t_0+T_s} = \left.\frac{\partial}{\partial q_k^j}\left(\frac{\partial \varphi_3}{\partial x_2}\right)\right|_{t=t_0+T_s+T_d} = 0,$$

and

$$\left.\frac{\partial}{\partial \lambda_1}\left(\frac{\partial \varphi_1}{\partial x_0}\right)\right|_{t=t_0} = \left.\frac{\partial}{\partial \lambda_1}\left(\frac{\partial \varphi_2}{\partial x_1}\right)\right|_{t=t_0+T_s} = \left.\frac{\partial}{\partial \lambda_1}\left(\frac{\partial \varphi_3}{\partial x_2}\right)\right|_{t=t_0+T_s+T_d} = 0.$$

Next, we slightly change the value of the control parameter, λ_2, and then repeat the same procedure to obtain a new bifurcation parameter value, λ_1^*. By repeating the procedure and gradually changing the value of λ_2, we can obtain a bifurcation set on the (λ_1, λ_2)-parameter plane. Figure S5A shows an example of two-parameter bifurcation

diagram of a periodic oscillation when the transcriptional response with the light adaptation defined by Eq. S1 under the LD cycles is incorporated to the autonomous circadian system defined by Eqs. 1-3. For the case of slow response of transcriptional response, a bifurcation diagram obtained by the above procedure is illustrated in Figure S5B. The lower and upper limits of the entrainment region shown in Figures 3-5 and Figure S2 were determined to calculate parameter values when the saddle-node and period-doubling bifurcations occur in the case where the period of the LD cycle was fixed as 24 h.
